# Response to comment by Moxon et al.

**DOI:** 10.1042/CS20171555

**Published:** 2018-01-02

**Authors:** Dawn Thompson, Nicola Morrice, Louise Grant, Samantha Le Sommer, Emma K. Lees, Nimesh Mody, Heather M. Wilson, Mirela Delibegovic

**Affiliations:** Institute of medical sciences, Aberdeen Cardiovascular and Diabetes Centre, School of Medicine, Medical Sciences and Nutrition, University of Aberdeen, Aberdeen, U.K.

We would like to thank *Clinical Science* for the opportunity to respond to the letter [1] which suggests that while we have been able to show that PTP1B inhibitor, trodusquemine, decreases atherosclerotic plaque size as well as serum triglycerides and cholesterol, that we have not shown that it reverses the plaque size, using *in vivo* imaging techniques such as MRI scanning or ultrasound.

**Figure 1:**
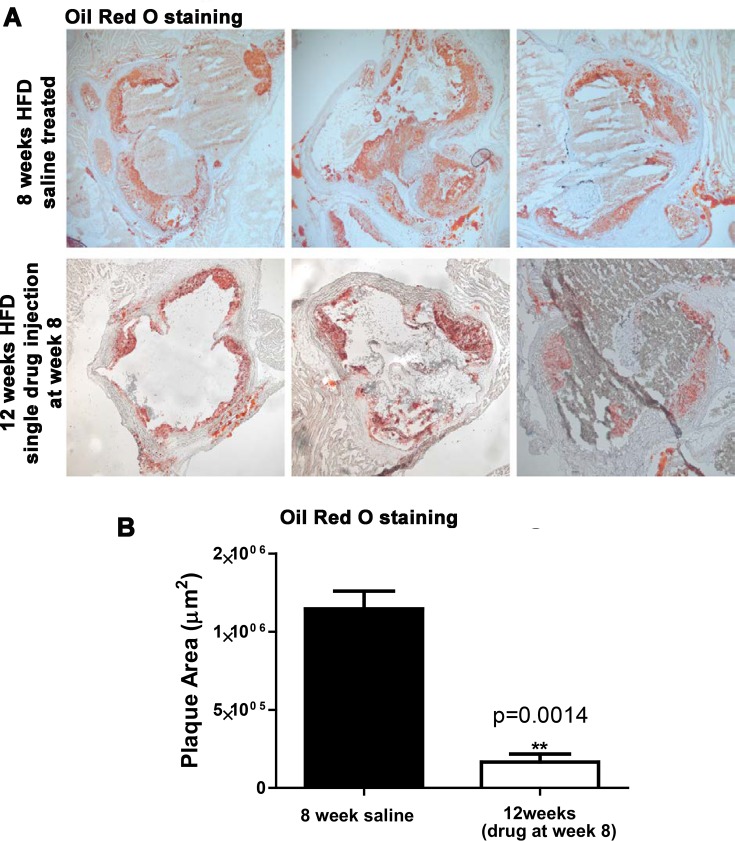
Global PTP1B inhibition effects on atherosclerotic plaque development (**A**) top panels: 8 weeks of HFD feeding stained with Oil Red O (n=3) versus bottom panel: 12 weeks HFD with single trodusquemine injection given at week 8 HFD (n=3); (**B**) quantification of plaque area as analaysed using Image J software. Data are represented as mean +/- S.E.M. and analysed by unpaired two-tailed t test where ** p=0.0014.

Both Ldrl^−/−^ and ApoE^−/−^ mouse models are historically, very well characterized mouse models of atherosclerosis that rapidly develop atherosclerotic plaques under high-fat/high-cholesterol dietary conditions. However, we wanted to confirm this in our own hands, using the gold-standard technique of sectioning the aorta and staining with Oil Red O over time. We now present data where we performed our pilot study to determine plaque load in these models, after 8 weeks of high-fat diet (HFD). As expected, at 8 weeks of HFD feeding, there was a well-established plaque deposition (Figure 1A,B). Our reversal study was therefore performed at 8 weeks HFD feeding, using a single injection of trodusquemine We demonstrate in our original article in Figure 5C,D [2], that indeed, we can reverse this established plaque.

Moreover, we recently reported that targeting PTP1B in the myeloid lineage cells alone on the ApoE^−/−^ background (LysM-PTP1B^−/−^/ApoE^−/−^) also protected against HFD-induced atherosclerotic plaque deposition and insulin resistance in the absence of any changes in body weight/adiposity [3], thus demonstrating in a set of genetic experiments, as well as pharmacological intervention with trodusquemine in Ldlr^−/−^ mice, that targeting PTP1B holds promise in atherosclerosis treatment and reduction in cardiovascular risk. It is also important to point out here that trodusquemine (also known as MSI-1436) has previously been shown to decrease food intake and body weight, decrease serum cholesterol and triglyceride levels and protect against hepatic steatosis in rodent models of obesity and diabetes [4–6].

While it would have been nice, in addition to the gold-standard well-established histological techniques used in our manuscript, to perform longitudinal *in vivo* studies cited in the letter [7], these do also suggest that the *in vivo* techniques are operator dependent (Table 2, Ref [7]). According to the literature and experts in the field, there is a general agreement that the tools to visualize plaques *in vivo* are rather limited in resolution, which is why there is currently an intense search for novel probes and improved imaging technologies. It would be nice in future experiments to use novel technologies such as 3D quantitation using optical project tomography [8] for the overall quantitative assessment but again this is a post-mortem technique not allowing longitudinal scanning. In our studies, by assessing the plaque load at 8 weeks of HFD, followed by pharmacological intervention, we can demonstrate that there is a reversal of a well-established plaque as stated in the manuscript. However, longitudinal studies and studies with improved techniques are needed to further confirm the findings presented.
